# An Overview of Clinical Oncology and Impact on Oral Health

**DOI:** 10.3389/froh.2022.874332

**Published:** 2022-04-25

**Authors:** Jack A. Harris, Giulia Ottaviani, Nathaniel S. Treister, Glenn J. Hanna

**Affiliations:** ^1^Department of Oral and Maxillofacial Surgery, Harvard School of Dental Medicine, Boston, MA, United States; ^2^Pathology, Lino Rossi Research Center, Department of Biomedical, Surgical and Dental Sciences, Università degli Studi di Milano, Milan, Italy; ^3^Division of Oral Medicine and Dentistry, Brigham and Women's Hospital, Boston, MA, United States; ^4^Department of Oral Medicine, Infection, and Immunity, Harvard School of Dental Medicine, Boston, MA, United States; ^5^Department of Medical Oncology, Dana-Farber Cancer Institute, Boston, MA, United States; ^6^Medicine, Harvard Medical School, Boston, MA, United States

**Keywords:** cancer, clinical oncology, oral health, oral complications, cancer therapy

## Abstract

As the incidence of cancer continues to increase, so too will the use of various forms of cancer therapeutics and their associated oral and dental complications. Although many of the acute and chronic oral toxicities of cancer therapy are largely unavoidable, appropriate and timely management of these complications has the potential to alleviate morbidity and improve outcomes. Undoubtedly, the substantial short- and long-term impacts of cancer therapy on the health of the oral cavity requires increased awareness, prevention, and treatment by multidisciplinary healthcare teams consisting of medical oncologists, dentists, and other oral healthcare specialists. This mini review provides a brief purview of the current state of clinical oncology and its impact on oral health. The topics introduced here will be further investigated throughout the remainder of the “Oral Complications in Cancer Patients” mini-review series.

## Introduction

Cancer accounted for roughly 10 million deaths in 2020, serving as a leading cause of mortality globally [1]. Cancer incidence is continuing to grow [2], reflecting population growth and aging, as well as the increasing prevalence of cancer risk factors associated with socioeconomic development. In the United States (US), half of men and one-third of women will develop cancer throughout their lifetime [3–5].

**Figure 1:**
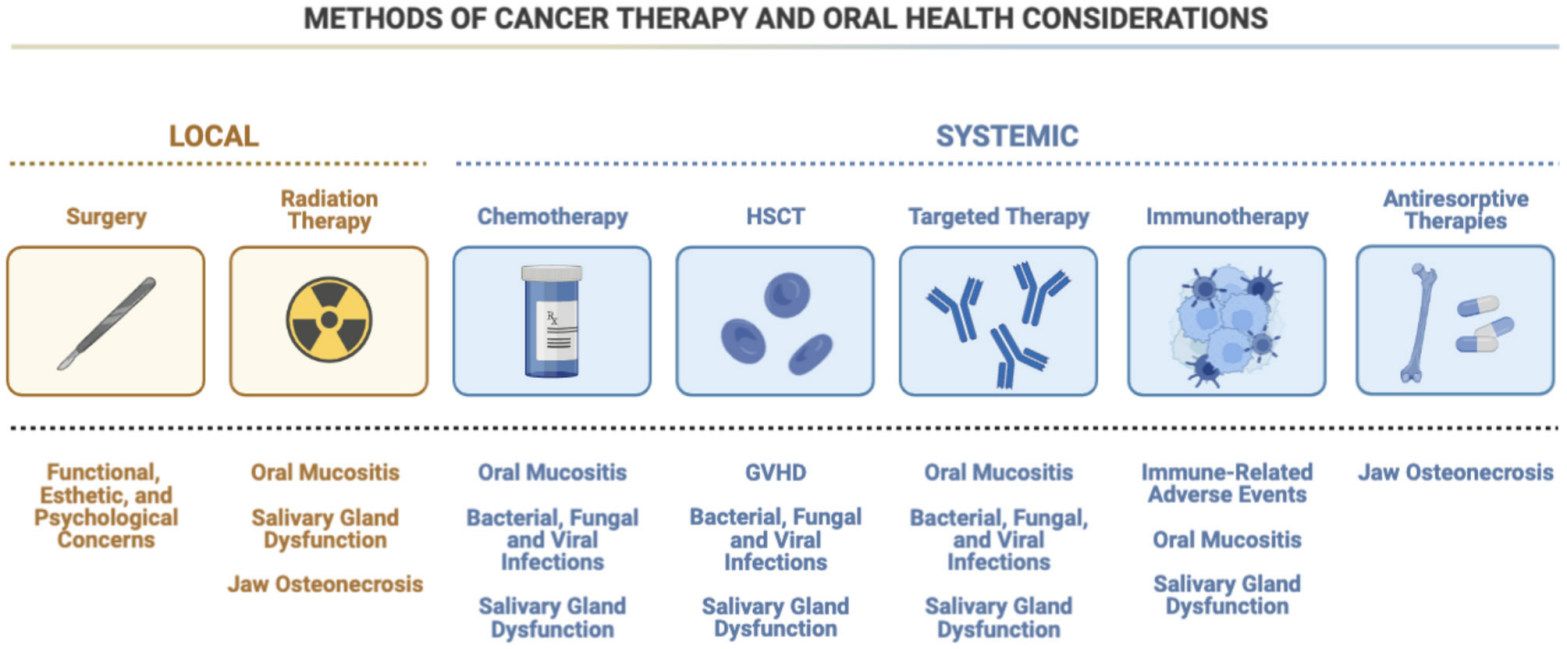
Methods of cancer therapy and oral health considerations. There are many types of possible therapies used to treat cancer, each with their own oral health side effects. For example, chemotherapy and targeted therapy designed to treat both local and systemic cancers carry with them similar concerns for oral mucositis, infection, and salivary gland dysfunction. Antiresorptive therapies may raise the possibility of osteonecrosis, which is also a concern when radiation therapy is used, although the possibility of emergent oral mucositis and salivary gland dysfunction are less prevalent with antiresorptive therapies. HSCT, hematopoietic stem cell transplantation; GVHD, graft-vs.-host disease; HSV, herpes simplex virus.

Many cancer treatment modalities, such as surgery, radiotherapy, chemotherapy (neoadjuvant, adjuvant, and/or concurrent), and hematopoietic stem cell transplantation, as well as supportive care measures (e.g., antiresorptive therapies) have the potential to cause various oral complications (Figure 1) [6]. More novel cancer therapeutics, such as targeted therapies [e.g., epidermal growth factor receptor (EGFR) inhibitors and tyrosine kinase inhibitors (TKI)] and emerging immunotherapies, have also demonstrated oral side effects [7–9]. As the incidence of cancer continues to increase, so too will the use of various forms of cancer therapeutics and their associated oral and dental complications.

This mini review provides a brief purview of the current state of clinical oncology and its impact on oral health. Clinical oncology consists of three primary disciplines: surgical oncology, radiation oncology, and medical oncology. Basic principles of clinical oncology, recent advancements in cancer therapeutics, and various oral health complications associated with cancer treatment will be discussed. Finally, the authors will consider various approaches to promoting oral health before, during, and after cancer treatment. The topics introduced here will be further investigated throughout the remainder of the “Oral Complications in Cancer Patients” mini-review series.

## Cancer Epidemiologic Trends

According to the Global Burden of Disease (GBD) study, cancer imposes the largest burden of any disease in the world, exceeding that of ischemic heart disease and stroke [10]. In 2018, over 18 million new cases of cancer were diagnosed; the most prevalent cancers among men were lung (1.37 million cases), prostate (1.28 million cases), and stomach (0.68 million), whereas women were most likely to be diagnosed with cancers of the breast (2.09 million cases), lung (0.72 million cases), and cervix/uterus (0.57 million cases) [11]. After ischemic heart disease, cancer remains the second leading cause of death worldwide, followed by stroke, and chronic obstructive pulmonary disease [12]. Over the last 15 years, the incidence of cancer has increased by 28%, which is 3-fold higher than the increase in mortality over the same period (~9%) [12]. Overall, individuals between the ages of 0–74 have a 10.6% risk of dying from cancer; men are most likely to die from lung, liver, and stomach cancer, whereas women are most likely to die from breast, lung, and cervix/uterus cancer [12]. By 2030, cancer is projected to be the leading cause of global mortality, surpassing that of ischemic heart disease [13].

Cancers of the head and neck (HNC) are a heterogeneous group of malignancies that comprise the ninth and seventh most common cancer in the US and world, respectively [2, 4]. Each year, head and neck squamous cell carcinoma (HNSCC) is diagnosed in over half a million patients and is responsible for over 380,000 deaths globally [14]. Oral squamous cell carcinoma (OSCC), a major concern among dentists, oral medicine providers, and other oral healthcare specialists, accounted for approximately 145,000 deaths worldwide in 2012 [15]. In the US, OSCC is responsible for roughly 3% of cancers in men and 2% of cancers in women, most of which are diagnosed after the age of 50 [16]. Five-year survival rates for OSCC are ~70%, although this number fluctuates substantially depending on anatomical/histologic subtype and grade/stage at the time of diagnosis [17].

## Cancer Therapy and Associated Oral Health Complications

### Surgical Management

Surgical management remains a mainstay of modern cancer treatment, including for HNSCC. While removal of simple, early stage tumors may result in minimal side effects, surgical treatment of more advanced stage lesions can produce numerous esthetic, functional, and psychological sequelae. Potential impacts of surgery on oral function include difficulty tasting, speaking, chewing, and swallowing, whereas the excision of mucosal surfaces, loss in soft tissue volume, and removal of bone may result in substantial esthetic deformities [18]. Maxillofacial prosthodontics is an essential component of oral rehabilitation in patients with oral cancer undergoing surgical management.

The primary goals of maxillofacial prosthodontics are to restore oral function, improve facial esthetics, and enhance quality of life [19]. For example, maxillofacial prosthodontists fabricate various appliances, such as obturators, partial bridges, etc., to support ongoing cancer treatment and for patients following surgical procedures [20]. By utilizing computed tomography imaging, medical modeling technology, and virtual surgical planning (VSP), maxillofacial prosthodontists are able to accurately pre-plan the prosthetic and dental rehabilitation of patients undergoing complex surgical reconstruction [21, 22]. Undoubtedly, the maxillofacial prosthodontist is an essential member of the cancer team and plays a vital role in achieving optimal treatment outcomes for cancer patients.

### Radiotherapy

Radiotherapy remains one of the primary treatment modalities for both localized and late-stage cancers. Further, various randomized control trials have demonstrated a superior tumor response to radiotherapy and concurrent chemotherapy, rather than radiotherapy alone, for numerous advanced tumors including HNCs [23–25]. The side effects and toxicities related to radiotherapy, however, have the potential to impart a substantial amount of morbidity and worsen quality of life for many patients. Despite recent advances in radiotherapy technique and delivery, many of the dental and oral complications related to head and neck radiation, such as oral mucositis, salivary gland dysfunction, radiation caries, and osteoradionecrosis, are still largely prevalent among this cancer population.

#### Oral Mucositis

Oral mucositis (OM) is fairly ubiquitous among patients receiving radiation therapy to the head and neck [26]. Symptoms of OM include irritation, discomfort, and pain that often precedes an erythematous mucosal lesion, which may ultimately progress to frank ulceration [27]. OM frequently occurs 2–3 weeks following high-dose radiation therapy to the head and neck (e.g., 60–70 Gy), and symptoms typically worsen with increasing radiation dose. Although any mucosal surface can potentially develop OM, non-keratinized tissues (e.g., buccal mucosa, lateral tongue, soft palate, and floor of mouth) are at a greater risk than keratinized tissues (e.g., attached gingiva, hard palate, dorsal tongue) [28]. The addition of concurrent chemotherapy or targeted therapies with radiation therapy has been shown to increase the severity, duration, and extent of OM [29]. The morbidity associated with OM includes pain, nutritional compromise often necessitating a feeding tube, reduction in quality of life, interruptions in cancer therapy, possible concomitant infections, and increased treatment costs [28]. Photobiomodulation, which uses red or near-infrared light to beneficially influence cellular metabolism to repair tissue damage caused by injury or disease, has been shown to be effective in preventing OM [30, 31].

#### Salivary Gland Dysfunction and Radiation Caries

Radiotherapy to the head and neck region is a common cause of salivary gland dysfunction, with doses of 50 Gy or higher imparting the highest risk for this complication [32, 33]. Patients may experience xerostomia in as little as 1 week after initiating radiation therapy, with the potential for permanent salivary gland dysfunction with continued exposure [34]. Disruption in the normal salivary flow may result in numerous oral complications, such as dysgeusia, dysphagia, problems with speech, oral candidiasis, and dental caries, while severely diminishing quality of life [35]. The enhanced risk of tooth decay, known as radiation caries, is thought to be a direct result of radiotherapy-induced salivary gland acinar degeneration and interstitial fibrosis [36, 37]. Teeth exposed to radiation may also be more prone to decay due to changes in the composition of dental hard tissue, such as loss of enamel prism structure, degeneration of odontoblast processes, and obliteration of dentinal tubules [38].

While various pharmacologic agents have been suggested as possible interventions for preventing radiation-induced salivary gland dysfunction, such as parasympathomimetic drugs, parasympatholytic drugs, and cytoprotective agents, the evidence to support their efficacy is of admittedly poor quality [34, 39–42]. Some clinicians and patients may opt for non-pharmacologic products such as toothpastes, mouthrinses, mouth sprays, and gels, as well as sugar-free gums and lozenges to reduce symptoms of xerostomia [43]. Fluoride supplementation, varnish, and regular oral hygiene check-ups are also essential to reduce the risk of developing radiation caries. In patients treated with radiotherapy, the daily application of 1% sodium fluoride gel has the potential to significantly reduce the incidence of caries [44].

#### Osteoradionecrosis

Osteoradionecrosis (ORN) of the jaw is a potentially severe iatrogenic disease of devitalized bone caused by radiation therapy of the head and neck that fails to heal or remodel [45–48]. Early proposed pathophysiologic mechanisms of ORN focused on hypoxic, hypovascular, and hypocellular tissue resulting in tissue breakdown and a non-healing wound [47, 49, 50]. More recent research, however, has favored the radiation-induced fibrosis theory whereby abnormal fibroblast activity leads to inflammation, local tissue injury, and eventually tissue necrosis [46, 51]. Common signs and symptoms of ORN include oral dysesthesia, paresthesia, pain, trismus, ulceration and necrosis of oral mucosa, malodor, pathologic fractures, draining fistulas, and deterioration in dental hygiene practices. Despite conflicting evidence, hyperbaric oxygen therapy (HBO) may be utilized in an attempt to prevent ORN of the jaws in adults receiving radiotherapy to the head and neck [52].

### Chemotherapy and Hematopoietic Stem Cell Transplantation

Chemotherapeutic agents comprise a vast group of chemicals designed to halt the growth of cancer cells, either through inducing apoptosis or preventing their replication. These agents produce their toxic effects by targeting rapidly proliferating cells, such as the basal cells of the mucosal layer as well as the acinar and ductal cells of the salivary glands [53]. The oral side effects of chemotherapy are relatively common and may include OM, candidiasis and other oral infections (including bacterial, viral, and fungal infections), xerostomia, oral bleeding, and potentially periodontal disease [53, 54]. Although concomitant chemotherapy often produces OM, the concomitant use of targeted agents may further alter mucositis risk, severity, and course [55, 56].

Hematopoietic stem cell transplantation (HSCT), on the other hand, involves the transplantation of healthy hematopoietic stem cells to patients with dysfunctional or depleted bone marrow for the treatment of various cancers, immune-deficiency syndromes, and hemoglobinopathies [57]. HSCT carries the risk of numerous acute and chronic complications that may impact the oral cavity, such as OM, oral candidiasis, herpes simplex virus (HSV) recrudescence, and graft-vs.-host disease (GVHD).

#### Graft-vs.-Host Disease

Graft-vs.-host disease (GVHD) is a major cause of morbidity and non-relapse mortality in patients undergoing allogeneic HSCT, with over 50% of patients developing chronic GVHD [58, 59]. Chronic GVHD is an alloimmune condition caused by donor T-cells recognizing and attacking antigens expressed on normal host tissues [60]. Oral chronic GVHD is characterized by mucosal, lichen planus-like changes presenting as erythematous and/or ulcerative lesions. Patients may experience oral pain, sensitivity to spicy/acidic foods, alcohol, and certain mouthwashes, xerostomia, difficulty speaking/swallowing, and taste changes that may predispose patients to decreased oral intake, nutritional deficiencies, and oral infections [61, 62]. Dentists and oral medicine specialists are important identifiers of this potentially debilitating disease. The most commonly used topical therapies for oral chronic GVHD include high-potency corticosteroids and calcineurin inhibitors, whereas systemic therapy includes corticosteroids, calcineurin inhibitors, and many other immunomodulatory agents [63].

### Antiresorptive and Antiangiogenic Therapy

Medication-related osteonecrosis of the jaw (MRONJ) is a potentially debilitating condition characterized by non-healing exposed bone in patients who have used either antiresorptive or antiangiogenic agents [64, 65]. High-dose regimens of antiresorptive medications, like bisphosphonates and receptor activator of nuclear factor kappa B ligand (RANKL) inhibitors (e.g., denosumab), are frequently used to prevent skeletal-related adverse events in adults with malignancies involving bone [66]. Although the pathophysiology of MRONJ has been greatly debated, most hypotheses suggest the role of altered bone remodeling, oversuppression of bone resorption, and angiogenesis inhibition as key mechanisms resulting in this disease process [67].

Dentists and other oral healthcare specialists play a vital role in preventing or minimizing a patient's risk of developing MRONJ. Dental assessments and the provision of prophylactic dental care prior to initiation of antiangiogenic or antiresorptive therapy have been shown to decrease a patient's chances of developing MRONJ [68]. In patients with MRONJ, treatment is divided into either conservative (such as maintaining optimal oral hygiene, eliminating soft and hard tissue disease, antibiotic therapy, and the use of antibacterial mouthwashes) or surgical management [68]. Although the success rate of surgical treatment for MRONJ has proven to be high [69], the side effects of these invasive resections have the potential to impart a substantial amount of morbidity.

### Targeted Therapies and Immunotherapies

Targeted therapies, which target specific genes and proteins involved in the growth and survival of cancer cells, and immunotherapies, which stimulate a patient's own immune system to combat cancer, are quickly becoming central pillars of cancer treatment. Although the introduction of targeted therapies and immunotherapies have revolutionized treatment for numerous types of malignancies, they have also produced novel side effects known as immune-related adverse events (irAE). While these adverse events have been observed in nearly all parts of the body, their impact on the oral cavity is notable [70].

#### Immune-Related Adverse Events

Although immunotherapy has made an indelible mark on the field of cancer therapeutics, irAEs associated with their use are unfortunately commonplace [71]. These adverse events are thought to arise from a loss of tolerance to self-antigens, which results in organ toxicity [72]. irAEs are found in nearly every organ, including the oral cavity where they primarily affect the oral mucosa, salivary glands, and sense of taste [73]. Numerous authors have reported an association between immunotherapy use and numerous oral complications, such as lichenoid reactions, sicca syndrome, vesiculobullous disorders, erythema multiforme (EM), and Steven-Johnson syndrome (SJS) [74–78]. Despite these observations, little is known regarding the etiology, underlying pathophysiology, and appropriate management for these conditions. Nevertheless, appropriate referrals to multidisciplinary teams composed of oncologists, rheumatologists, and oral healthcare specialists should be made to ensure proper case management for this complex patient population.

## Promoting Oral Health With Cancer Treatment

### Before Cancer Treatment

Obtaining dental clearance is an essential step prior to the initiation of cancer treatment, particularly for patients to be exposed to radiation to the head and neck. The rationale for dental screening prior to cancer therapy derives from numerous studies linking an increased incidence of intra-therapy complications, such as acute dental infections, with poor oral health [79–81].

A complete oral evaluation prior to the initiation of cancer treatment should elucidate the following information: presence of a dental home, date of last dental visit, recognition of past and current dental problems, an oral/dental evidence-based risk assessment such as CAMBRA (caries management by risk assessment), and an evaluation of past and current medication use.

Additionally, the patient should receive oral prophylaxis including professional hygiene therapy as well as eliminate any existing low-grade infections and possible sources of trauma (e.g., trauma from denture or fixed orthodontic appliances) [82]. The National Institute for Dental and Craniofacial Research recommends that elective surgical procedures be postponed until the cessation of cancer therapy, while invasive procedures be completed at least 14 days prior to the initiation of head and/or neck radiation and 7–10 days prior to myelosuppressive chemotherapy [83]. Finally, patients should be educated in proper oral hygiene techniques for the prevention of future dental caries and oral disease that may impact cancer treatment.

### During Cancer Treatment

The increased risk of acute oral complications resulting from radiation and/or chemotherapy highlights the importance of continued proper oral hygiene practices and maintenance. Patients should continue to use the modified Bass brushing method with fluoride toothpaste at least two times per day [84], floss one time per day, avoid the use of alcoholic mouthwashes, and remain on a regular hygiene recall schedule with their dentist. The use of removable appliances and prostheses, especially those with the potential to cause hard and soft tissue damage, should be limited. Stimulation of salivary production in those patients demonstrating hyposalivation can be achieved either through the use of sugar-free lozenges or gums (e.g., xylitol) [85, 86].

It is crucial that dentists and other oral healthcare specialists maintain communication with the oncologist throughout the duration of cancer therapy and obtain proper consultation with the oncologist prior to any dental procedures, including prophylaxis. A blood sample should be obtained from patients undergoing chemotherapy roughly 24 h prior to any invasive oral surgical procedures and postponed when the following are present: platelet counts <75,000/mm^3^; abnormal clotting factors present; and/or absolute neutrophil count of <1,000/mm^3^ [83].

### After Cancer Treatment

Following the completion of cancer therapy, patients should remain on a regular recall schedule as recommended by their dentist and continue practicing proper oral hygiene including the use of fluoride toothpaste and varnish. In patients at higher risk for dental caries (especially post-allogeneic HSCT/GVHD) as well as those with a history of oral malignancy (such as OSCC), a more intensive and frequent recall schedule may be necessary. Further, a patient's hematologic status, such as the resolution of immunosuppression and/or thrombocytopenia following the cessation of cancer therapy, should be assessed prior to dental treatment. Furthermore, the dentist should update the patient's medication list and assess for any anti-resorptive and/or anti-angiogenic medications used throughout the duration of cancer treatment.

Finally, childhood cancer therapy has the potential to result in numerous dental, craniofacial, and soft tissue complications. Children are particularly susceptible to the long-term effects of cancer therapy as treatment typically occurs during the most active stage of growth and organ development [87]. For example, these patients are at an increased risk for dental caries, abnormalities in tooth morphology and composition, hyposalivation, maxillary and mandibular growth disturbances, and temporomandibular dysfunction (TMD) [88, 89]. As such, pediatric and general dentists should be aware of these potential complications and monitor for any abnormal deviations in craniofacial and/or dental growth and development [83].

## Conclusion

Innovations and improvements in cancer therapy have substantially increased survivorship in recent years. As a result, there is a growing need for continuing management of the oral health needs of this population. Although many of the acute and chronic oral toxicities of cancer therapy are largely unavoidable, appropriate and timely management of these complications has the potential to alleviate a considerable amount of morbidity. Further, with advances in computational modeling and “Deep Learning” protocols, individuals at risk for developing drug toxicities may be identified and providers may be better equipped to predict which patients and drugs are most likely to induce oral side effects. Finally, the successful management of this complex patient population requires interprofessional collaboration and the utilization of a comprehensive, patient-centered approach with an emphasis on oral health.

## Author Contributions

JH and GO wrote the first draft of the manuscript. GH guided and revised the manuscript. NT conceptualized and guided the project. All authors contributed to the article and approved the submitted version.

## Funding

GO was supported in part by the Piano di Sostegno alla Ricerca (PSR) 2020, Linea 2: Dotazione Annuale per attività istituzionali, Department of Biomedical, Surgical and Dental Sciences, Università degli Studi di Milano, Milan, Italy.

## Conflict of Interest

The authors declare that the research was conducted in the absence of any commercial or financial relationships that could be construed as a potential conflict of interest.

## Publisher's Note

All claims expressed in this article are solely those of the authors and do not necessarily represent those of their affiliated organizations, or those of the publisher, the editors and the reviewers. Any product that may be evaluated in this article, or claim that may be made by its manufacturer, is not guaranteed or endorsed by the publisher.
